# Pipeline embolization device-assisted angioplasty for type II proatlantal intersegmental artery dissection inducing an embolic shower

**DOI:** 10.3389/fneur.2025.1490799

**Published:** 2025-02-04

**Authors:** Li Bao, Zhenguo Pan, Haifeng Ji, Shuang He

**Affiliations:** ^1^Department of Stroke Center, Affiliated Hospital of Nantong University, Nantong, China; ^2^Medical College of Nantong University, Nantong, China; ^3^Department of Neurology, Xiangshui People’s Hospital, Yancheng, China

**Keywords:** proatlantal intersegmental artery, pipeline embolization device, dissection, stroke, angioplasty, HR-VWI

## Abstract

The proatlantal intersegmental artery (PIA) plays a crucial role in blood supply during embryonic development, and failure of its closure can lead to the persistent proatlantal intersegmental artery (PPIA), which may result in pathological changes such as dissection and aneurysms. We present a case of a patient with right type II PPIA dissection inducing an embolic shower, accompanied by left vertebral artery hypoplasia (VAH). Digital subtraction angiography (DSA) and high-resolution magnetic resonance vascular wall imaging (HRMR-VWI) showed the aneurysmal dilation of the false lumen in the right PPIA dissection and indicated the high risk of mural thrombosis and dislodgement. Following a comprehensive evaluation of the patient’s condition, we conducted pipeline embolization device (PED)-assisted angioplasty to treat the PPIA dissection and mitigate the risk of recurrent strokes. Postoperative follow-up indicated that the patient recovered smoothly, with no signs of recurrent stroke. This case highlights the critical need for prompt recognition and intervention in cases of rare vascular variants. The flow diverter implantation can greatly enhance patient outcomes and lower the risk of recurrent strokes, offering important insights for the clinical management of similar cases. Additional research is necessary to investigate the underlying pathological mechanisms of PPIA and its connection to stroke occurrence, which will help refine treatment strategies in the future.

## Introduction

In embryonic development, the primitive carotid-vertebrobasilar anastomoses facilitate blood flow from the primitive internal carotid artery (ICA) to the posterior circulation. There are four types of carotid-vertebrobasilar anastomoses: the primitive trigeminal, otic, hypoglossal and proatlantal intersegmental artery (PIA) (1). The regression of these anastomotic channels begins as the embryo reaches a size of 7 to 12 mm, with the PIA being the last one (1, 2). In cases where anastomotic closure does not occur, these vessels persist into adulthood (2, 3). Among these, the persistent proatlantal intersegmental artery (PPIA) represents the rarest type of persistent carotid-vertebrobasilar anastomosis in adults, often identified incidentally (4). The PPIA originates from the common carotid artery (CCA), external carotid artery (ECA), or ICA, and penetrates the cranial cavity via the foramen magnum of the occipital bone, converging with the V3 segment of the vertebral artery (VA). There are two primary variants of PPIA: type I, originating from the ICA, and type II, more frequently arising from the ECA (5, 6).

This report examines a rare instance of a right type II PPIA dissection, which repeatedly inducing an embolic shower (ES). We determined that the ES were caused by thrombus dislodgement from the false lumen of the PPIA dissection. Given the ineffectiveness of pharmacological treatment, we performed PPIA angioplasty using a pipeline embolization device (PED), aiming to prevent ES caused by thrombus dislodgement.

## Case

A 53-year-old male with a history of paroxysmal dizziness and hypertension experienced a sudden exacerbation of dizziness and onset of left hemiparesis. Initial evaluation at a local hospital showed stable vital signs and unremarkable laboratory findings. Cranial magnetic resonance imaging (MRI) revealed an acute infarction in the right brainstem and right cerebellum, with encephalomalacia in the right thalamus (Supplementary Figures S1A–C). Magnetic resonance angiography (MRA) indicated left VA agenesis (Supplementary Figure S1D). The patient was diagnosed with multiple lacunar strokes (LS) and received conservative treatment including dual antiplatelet therapy (aspirin and clopidogrel), lipid-lowering agents (statins), and neuroprotective medications (edaravone and N-butylphthalide). Computed tomography (CT) 1 week later revealed a hypodense lesion in the right cerebellum (Supplementary Figure S1E).

Despite ongoing antiplatelet therapy, 2.5 months after initial stroke the patient developed symptoms of stroke, including bilateral blurred vision, facial asymmetry, and unsteady gait, after waking from a nap on the morning. CT scan revealed lacunar infarction and encephalomalacia in the right cerebellum and right thalamus (Supplementary Figures S2A,B). MRI suggested new infarct foci in the right cerebellum and left thalamus, alongside the earlier encephalomalacia in the right cerebellum and right brainstem (Supplementary Figures S2D–F). MRA once again confirmed left VA agenesis (Supplementary Figure S2C). Given the contraindication for intravenous thrombolysis owing to the patient’s recent stroke history, the treating team opted to continue with conservative management.

**Figure 1:**
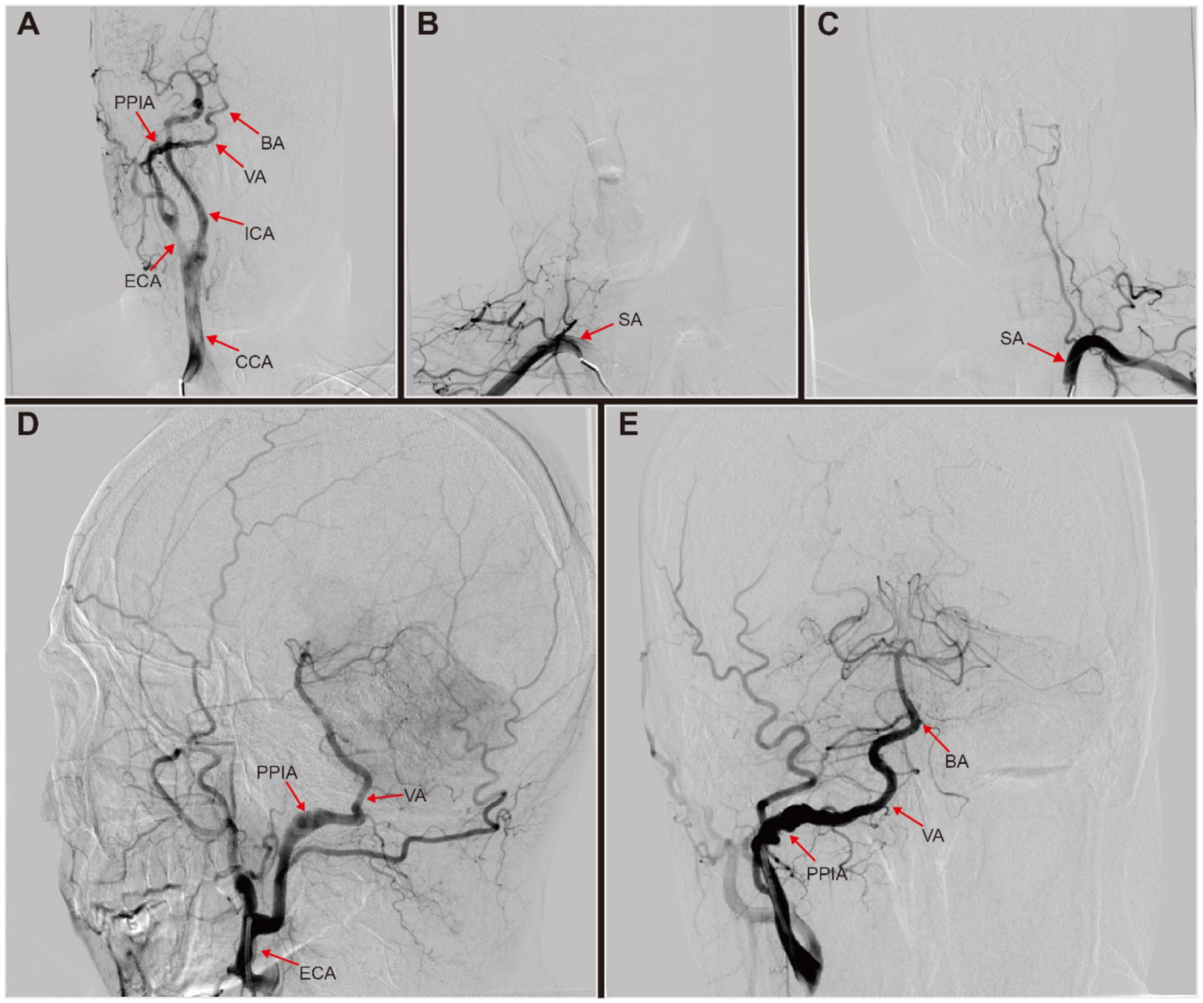
Digital subtraction angiography (DSA) of this patient with right persistent proatlantal intersegmental artery (PPIA) dissection. **(A)** Right common carotid artery (CCA) injection revealed a prominent type II PPIA branching off from the right external carotid artery (ECA). This artery connected to the ipsilateral vertebral artery (VA), supplying basilar artery (BA). **(B)** Right subclavian artery (SA) injection showed no origin of the right VA from the SA. **(C)** Left SA injection revealed left VA hypoplasia with no visualization of BA. **(D,E)** Right ECA injection revealed a right PPIA with dissection aneurysmal dilatated, which connected to the right VA to supply the BA.

Following stabilization, the patient was transferred to our institution 19 days after second stroke to elucidate the etiology of recurrent LS and to improve his prognosis. A digital subtraction angiogram (DSA) was performed to assess the cerebral vasculature. The DSA revealed a branch from the right external carotid artery (ECA) merging into the ipsilateral vertebral artery (VA), absent VA branching from the right subclavian artery, and left vertebral artery hypoplasia (VAH) with no significant abnormalities in the remaining vessels (Figures 1A–E). Further review of DSA led to the diagnosis of a right type II persistent proatlantal intersegmental artery (PPIA) dissection with an aneurysmal dilatation in the false lumen, alongside left VAH. After ruling out atrial fibrillation by 24-h ambulatory electrocardiographic monitoring, we hypothesized that mural thrombus dislodgement from the PPIA dissection was the probable cause of the ES observed in this patient.

**Figure 2:**
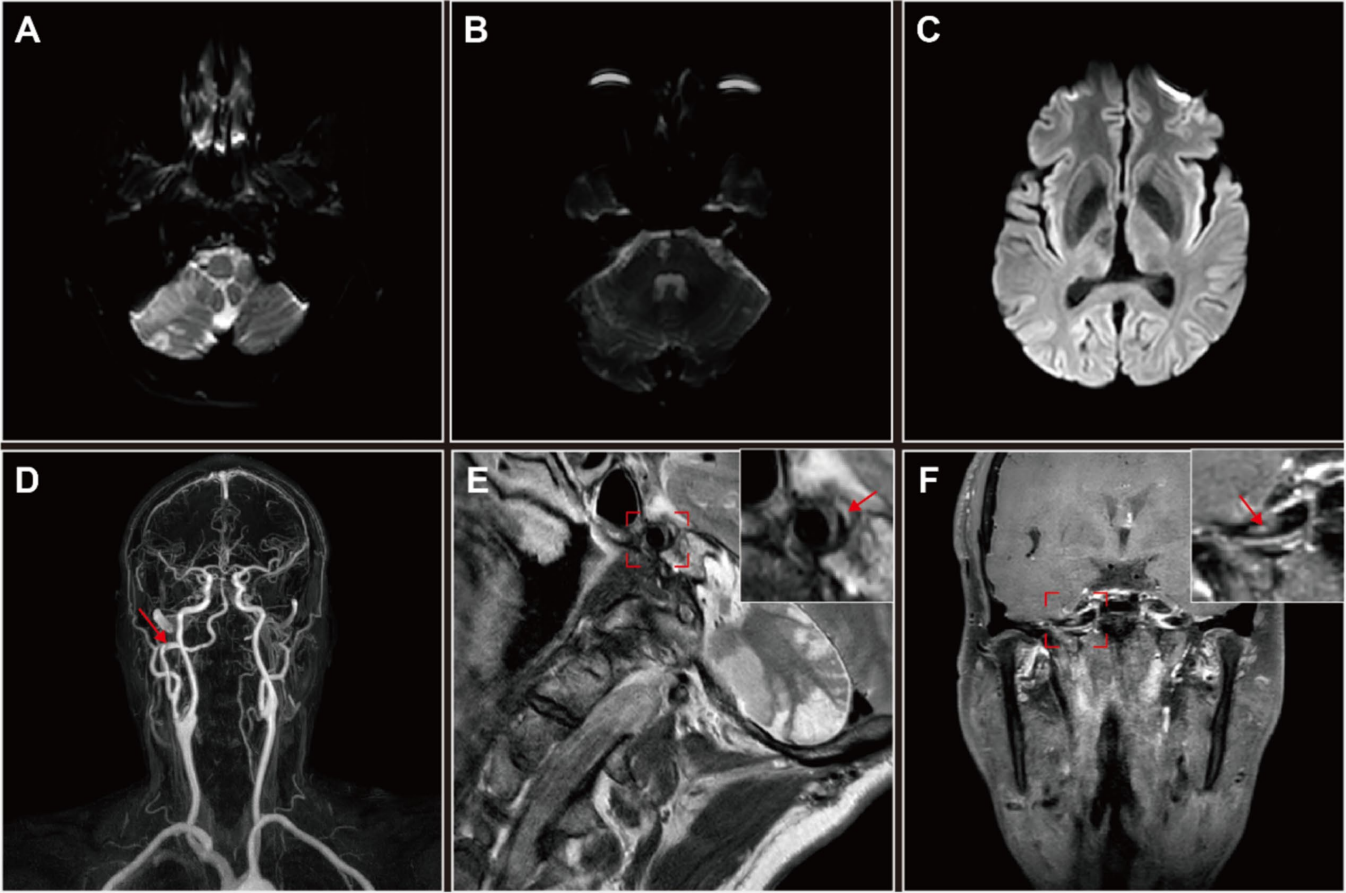
High-resolution magnetic resonance imaging (HRMRI) of the head and neck in this patient with right persistent proatlantal intersegmental artery (PPIA) dissection. **(A–C)** Diffusion weighted imaging (DWI) revealed multiple infarct foci and encephalomalacia across various cerebral regions: the right cerebellum **(A)**, right brainstem **(B)** and right thalamus **(C)**. **(D)** MRA suggested an dissection aneurysmal dilatation of the right PPIA, which is the exclusive supplier to the vertebrobasilar artery system. **(E,F)** High-resolution magnetic resonance vascular wall imaging (HRMR-VWI). **(E)** Sagittal contrast-enhanced T2-weighted imaging revealed eccentric thickening of the PPIA arterial wall accompanied by a crescent-shaped subacute-phase intramural hematoma (arrow) in the thickened lumen wall. The detailed sagittal profile of the PPIA, highlighted in a red frame, is positioned in the upper right corner. **(F)** Coronal contrast-enhanced T1-weighted imaging revealed inhomogeneous thickening of the intimal flap of the PPIA dissection (arrow), suggesting irregular attachment of fibrous tissue. The detailed coronal profile of the PPIA, encased in a red frame, is situated in the upper right corner. Both coronal and sagittal HRMR-VWI indicated a right PPIA dissection aneurysmal dilatation and a high risk of mural thrombosis and dislodgement.

To confirm this diagnosis, high-resolution magnetic resonance imaging (HRMRI) was performed, which indicated infarct and encephalomalacia in the right cerebellum, right brainstem, and right thalamus (Figures 2A–C). MRA corroborated the presence of a right type II PPIA dissection with contralateral VAH, consistent with DSA findings (Figure 2D). High-resolution magnetic resonance vascular wall imaging (HRMR-VWI) displayed a subacute intramural hematoma and intimal flap thickening in the dissection’s false lumen, which were considered high-risk factors for mural thrombosis and dislodgement (Figures 2E,F).

**Figure 3:**
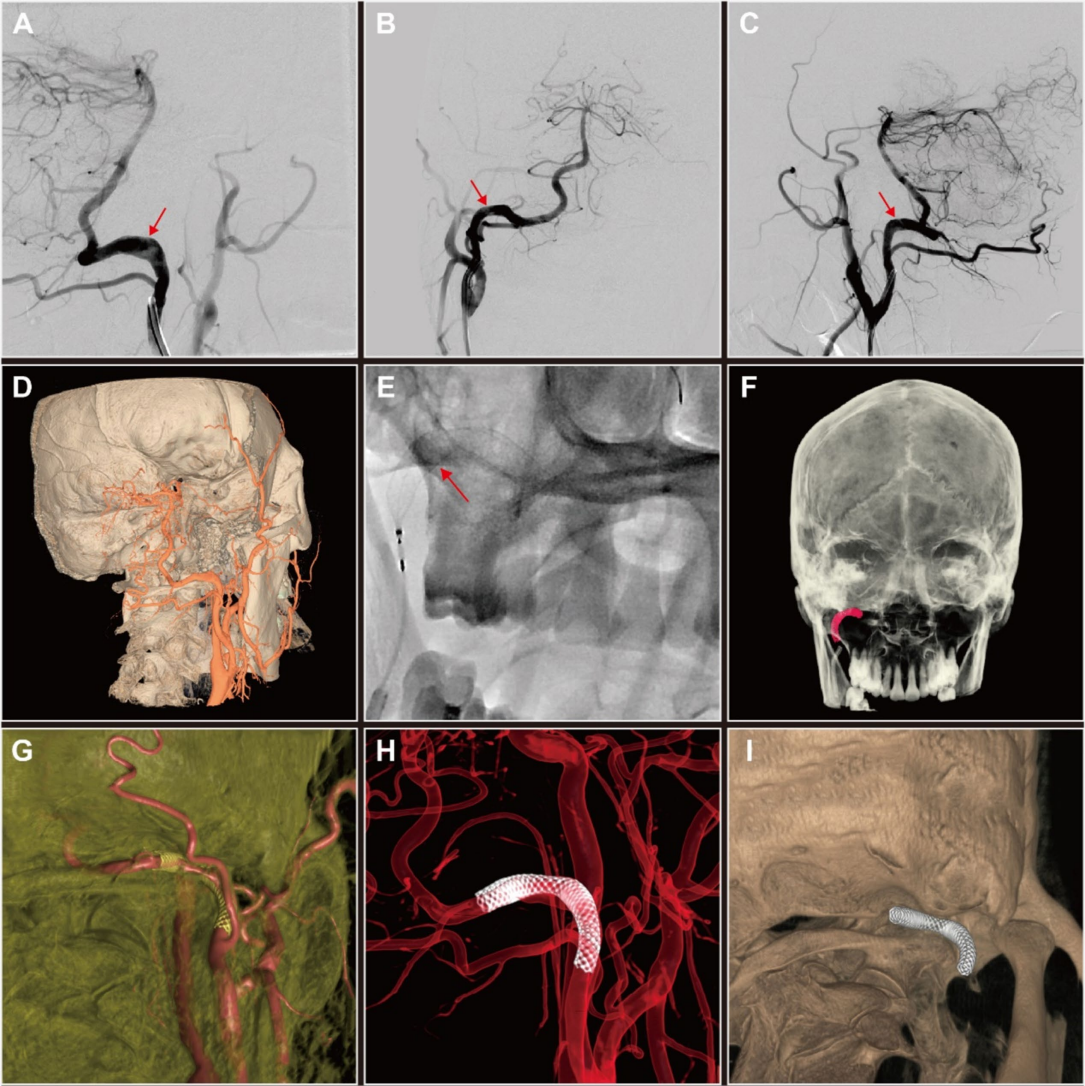
Digital subtraction angiography (DSA) and three-dimensional (3D) reconstruction of persistent proatlantal intersegmental artery (PPIA) angioplasty with flow diverter implantation of this patient with right PPIA dissection. **(A–C)** Comparative DSA images of the right PPIA dissection (arrow) pre-implantation **(A)** and post-implantation **(B,C)** of pipeline embolization device (PED). **(D)** DSA-based 3D reconstruction of PPIA dissection prior to PED implantation. **(E)** Release of PED. **(F–I)** Post-implantation DSA-based 3D reconstruction of PED in the PPIA highlighted the improvements in vascular architecture and the therapeutic benefits of the intervention.

Given the patient’s history and HRMR-VWI results, we concluded that addressing the right type II PPIA dissection could mitigate the risk of the recurrent ES. Given that the true lumen stenosis at the site of the dissection is not severe, we chose to perform simple PED implantation for PPIA angioplasty. The PED (4.5 mm × 30 mm; Flex, Medtronic, United States) was successfully delivered and deployed under the guidance of the microcatheter (Phenom27, Medtronic, United States) and microwire (Synchro2, Stryker, United States) after the guide catheter (Envoy, Codman, United States) was in position (Figure 3E). Postoperatively, three-dimensional reconstructions of the DSA images demonstrated the PED effectively reshaping the PPIA, isolating the aneurysmal dilated false lumen, and securing posterior circulation blood flow (Figures 3A–I).

**Figure 4:**
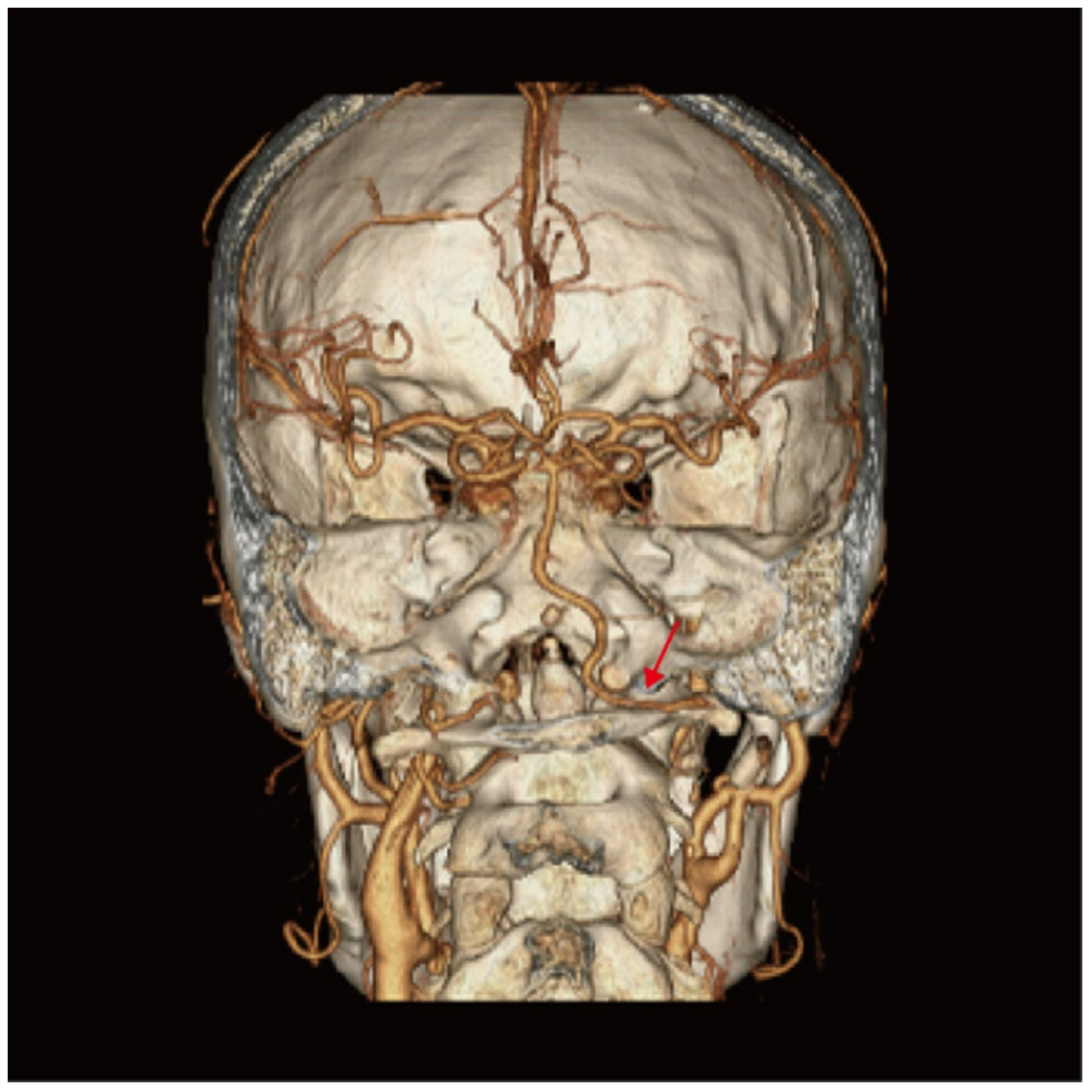
Computed tomography angiography (CTA) of the patient 1 month after persistent proatlantal inter-segmental artery (PPIA) angioplasty with flow diverter implantation. Right PPIA (arrow) aneurysmal dilatation was significantly improved compared to the pre-intervention images.

One month post-procedure, the patient reported no new symptoms. A repeat cranial CTA demonstrated significant reduction in the aneurysmal dilatation of the right PPIA dissection and optimal remodeling of the PPIA, compared to previous images (Figure 4).

## Discussion

By the end of the sixth week of gestation, when the embryo has developed to 12–14 mm, the PIA physiologically regresses completely, leading to the formation of the VA (7). However, poor anastomotic closure can result in persistent patency of the primitive PIA, transforming into a PPIA, and hypoplasia of the ipsilateral VA (3). Embryologically, VA is constructed through the fusion of multiple longitudinal anastomoses between neighboring cervical intersegmental arteries (8). Difficulty in fusion between any of the segments of the VA under the influence of various congenital or acquired factors that impede VA formation could also lead to persistent opening of the ipsilateral PIA to ensure posterior circulatory blood supply. A notable case report by Zuflacht et al. (9) illustrated a unique unilateral type II PPIA functioning in conjunction with the ipsilateral CCA to sustain whole brain blood flow.

PPIA has been implicated in a variety of vascular pathologies, albeit infrequently. It has been reported that type I PPIA could cause top of the basilar syndrome (10), pontine infarction (11), cerebral watershed and posterior circulation infarction (12). Type II PPIA could cause dizziness and syncope (13), transient hand weakness and amaurosis fugax when occurring with severe ICA stenosis (14). The linkage between PPIA, VAH, and intracranial aneurysms has been extensively documented, highlighting a prevalent association with vascular anomalies (5, 15, 16).

In the present case, diagnostic exclusions were made for atherosclerotic and cardioembolic cerebral infarction based on an infarct diameter of less than 1.5 cm, absence of atherosclerotic vulnerable plaques and vessels with no greater than 50% stenosis on DSA and HRMR-VWI, as well as the absence of atrial fibrillation in this patient (17). A right type II PPIA with a dissection characterized by aneurysmal dilatation of the false lumen, elevating the risk of mural thrombosis. This pathology was identified as the primary cause of the ES in a 53-year-old patient. Given the high-risk nature of the pathology, characterized by subacute intramural hematoma and extensive fibrous tissue attachment to the intimal flap, and the ineffectiveness of conservative drug treatments, we preferred PED implantation to avoid thrombus formation and dislodgement. Follow-up neurologic function assessment, CTA and CT Perfusion (CTP) showed that the PED effectively maintained posterior circulation, and the patient experienced no recurrent ischemic strokes.

This case is the first documented instance of a type II PPIA dissection leading to an ES. The type II PPIA dissection was initially identified on DSA as originating from the ECA and displayed aneurysmal dilatation changes before traversing the occipital foramen. Although intravascular imaging techniques such as MRA and DSA are adept at visualizing lumen morphology, they fall short in detecting dissection lesions with normal vessel diameters or fully delineating the aneurysmal wall. The unique capabilities of HRMR-VWI allowed for the detection of likely thrombosis formation and dislodgement within the dissected PPIA, confirming its role as the causative lesion.

In general, early and precise identification of vascular variants like PPIA is crucial in clarifying the clinical diagnoses, underlying etiologies, and therapeutic options. While PPIA is often linked with serious posterior circulation strokes and other cerebrovascular pathologies, it can also facilitate critical interventions such as stent thrombectomy (18), angioplasty (19), and reflux compensation (20), occasionally proving to be lifesaving.

## Data Availability

The original contributions presented in the study are included in the article/Supplementary material, further inquiries can be directed to the corresponding author.

## References

[1] Vasović L Mojsilović M Anđelković Z Jovanović I Arsić S Vlajković S. Proatlantal intersegmental artery: a review of normal and pathological features. Childs Nerv Syst. (2009) 25:411–21. doi: 10.1007/s00381-008-0765-7, PMID: 19212779

[2] Purkayastha S Gupta AK Varma R Kapilamoorthy TR. Proatlantal intersegmental arteries of external carotid artery origin associated with Galen’s vein malformation. AJNR Am J Neuroradiol. (2005) 26:2378–83. PMID: 16219849 PMC7976148

[3] Luh GY Dean BL Tomsick TA Wallace RC. The persistent fetal carotid-vertebrobasilar anastomoses. AJR Am J Roentgenol. (1999) 172:1427–32. doi: 10.2214/ajr.172.5.10227532, PMID: 10227532

[4] Dimmick SJ Faulder KC. Normal variants of the cerebral circulation at multidetector CT angiography. Radiographics. (2009) 29:1027–43. doi: 10.1148/rg.294085730, PMID: 19605654

[5] Dimitriade A Stănciulescu R Dorobăţ B Iana G. A symptomatic presentation of a rare type of proatlantal artery. Diagn Interv Imaging. (2016) 97:371–2. doi: 10.1016/j.diii.2015.11.002, PMID: 26724857

[6] Zarghouni M Marichal D. Persistent bilateral proatlantal type II artery. Proc (Bayl Univ Med Cent). (2013) 26:50–1. doi: 10.1080/08998280.2013.11928918, PMID: 23382615 PMC3523771

[7] Yilmaz E Ilgit E Taner D. Primitive persistent carotid‐basilar and carotid‐vertebral anastomoses: a report of seven gases and a review of the literature. J Neurosurg. (1995) 8:36–43. doi: 10.1002/ca.980080107 7697511

[8] Padget DH. Designation of the embryonic intersegmental arteries in reference to the vertebral artery and subclavian stem. Anat Rec. (1954) 119:349–56. doi: 10.1002/ar.1091190306, PMID: 13197795

[9] Zuflacht JP Liang C Burkhardt JK Favilla CG. Whole-brain perfusion via right common carotid artery with type 2 proatlantal intersegmental artery. Stroke. (2022) 53:e481–2. doi: 10.1161/strokeaha.122.040239, PMID: 35959680

[10] Bahşi YZ Uysal H Peker S Yurdakul M. Persistent primitive proatlantal intersegmental artery (proatlantal artery I) results in ‘top of the basilar’ syndrome. Stroke. (1993) 24:2114–7. doi: 10.1161/01.str.24.12.2114, PMID: 8248997

[11] Ikeda M Okamoto K Hirai S Amari M Takatama M. A case of pontine infarction with persistent primitive proatlantal artery. Rinsho Shinkeigaku. (1993) 33:976–9. PMID: 8299279

[12] Schoof J Skalej M Halloul Z Wunderlich MT. Carotid endarterectomy in a patient with persistent proatlantal artery. Cerebrovasc Dis. (2007) 23:458–9. doi: 10.1159/000101748, PMID: 17435385

[13] Gocer GC Ogul H. Unusual vascular cause of syncope in an adult patient: type 2 proatlantal intersegmental artery. Acta Neurol Belg. (2024) 124:1671–3. doi: 10.1007/s13760-024-02538-5, PMID: 38565762

[14] Liechty JM Weddle RJ Shutze WP Smith BL. Occurrence of a type 2 proatlantal intersegmental artery during carotid endarterectomy for symptomatic stenosis. J Vasc Surg. (2016) 64:807–8. doi: 10.1016/j.jvs.2014.10.100, PMID: 27565597

[15] Kolbinger R Heindel W Pawlik G Erasmi-Körber H. Right proatlantal artery type I, right internal carotid occlusion, and left internal carotid stenosis: case report and review of the literature. J Neurol Sci. (1993) 117:232–9. doi: 10.1016/0022-510x(93)90178-2, PMID: 8410060

[16] Nonaka Y Nakatani K Tanigawara T Hattori T Ohkuma A Kaku Y. A case of a persistent primitive proatlantal intersegmental artery with a ruptured basilar bifurcation aneurysm. No Shinkei Geka. (2001) 29:775–9. PMID: 11554097

[17] Neurology Society of Chinese Medical Association. Diagnostic criteria of cerebrovascular diseases in China. Chin J Neurol. (2019) 52:710–5. doi: 10.3760/cma.j.issn.1006-7876.2019.09.003

[18] Zhao L Yang L Liu X Wang X Zhang G Wu J. Case report: Stent retriever thrombectomy of acute basilar artery occlusion via the type 1 proatlantal intersegmental artery. Front Neurol. (2022) 13:812458. doi: 10.3389/fneur.2022.812458, PMID: 35677331 PMC9168035

[19] Babici D Johansen PM Sial N Snelling B. Intracranial angioplasty via type II proatlantal intersegmental artery. Cureus. (2023) 15:e47724. doi: 10.7759/cureus.47724, PMID: 38021511 PMC10676235

[20] Haiqi Z Feng L Cong L. Reflux compensation of persistent proatlantal intersegmental artery: report of one case. Br J Neurosurg. (2023) 37:1154–6. doi: 10.1080/02688697.2020.1850639, PMID: 33263438

