# Supplementary figures and images for: Pipeline embolization device-assisted angioplasty for type II proatlantal intersegmental artery dissection inducing an embolic shower
Source: Front Neurol. 2025 Feb 4;16:1490799. doi: 10.3389/fneur.2025.1490799 (PMC11832404; doi:10.3389/fneur.2025.1490799)

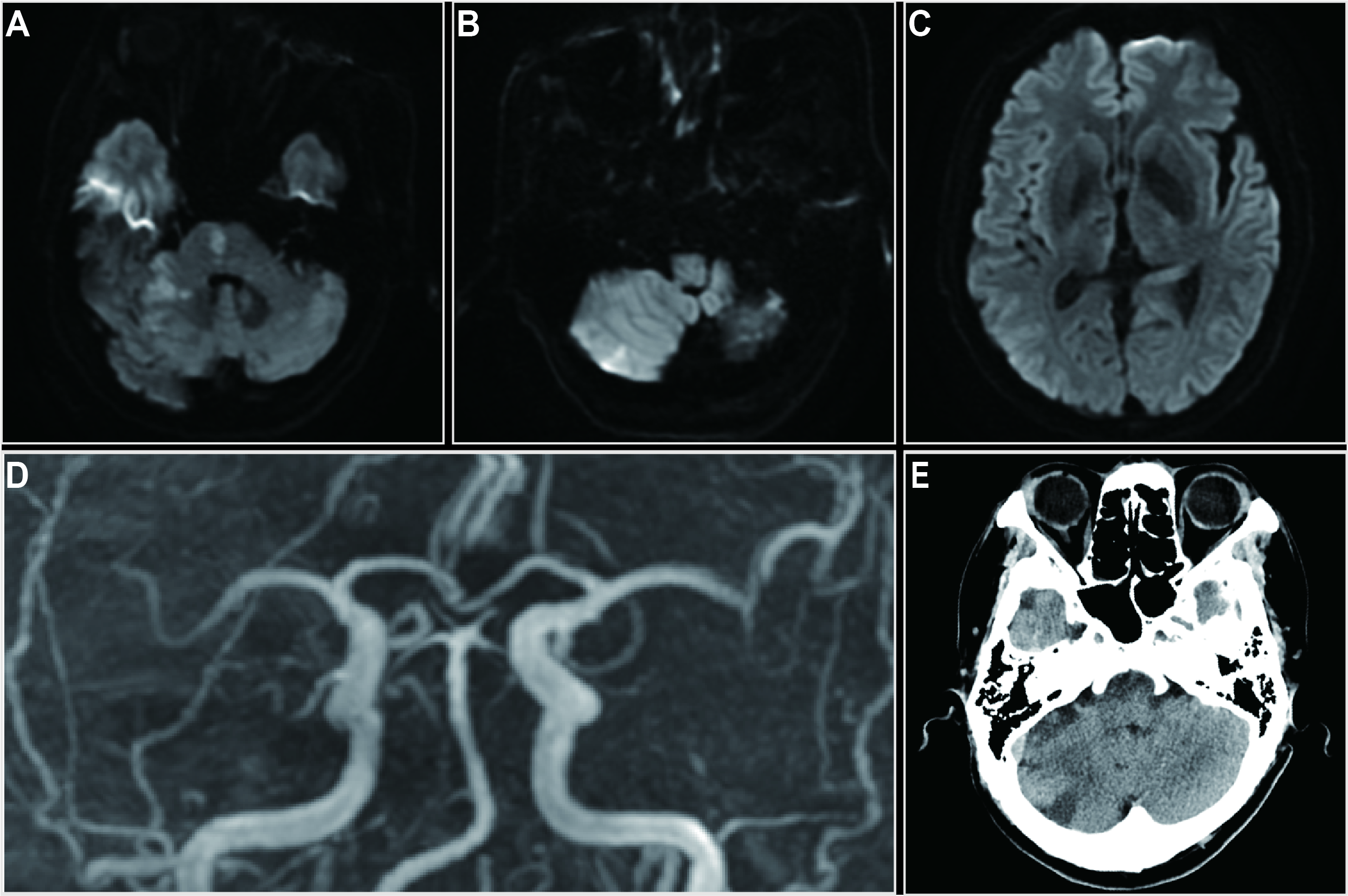

Supplement: Supplementary file 1 [file Image_1.TIF]

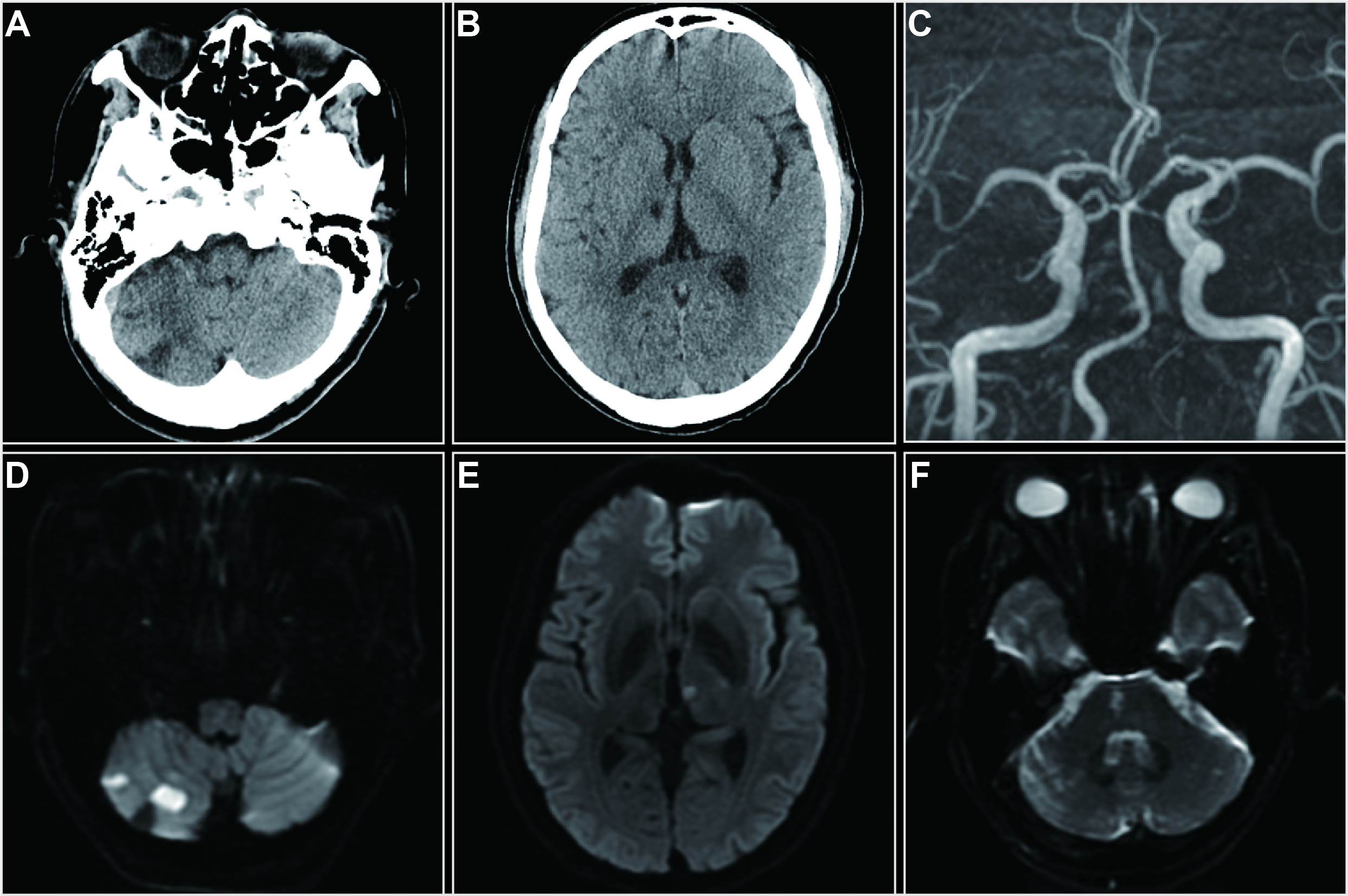

Supplement: Supplementary file 2 [file Image_2.TIF]
